# How long do rapid diagnostic tests remain positive after anti-malarial treatment?

**DOI:** 10.1186/s12936-018-2371-9

**Published:** 2018-06-08

**Authors:** Ursula Dalrymple, Rohan Arambepola, Peter W. Gething, Ewan Cameron

**Affiliations:** 1 Department of Zoology, University of Oxford, New Radcliffe House, Radcliffe Observatory Quarter, Woodstock Rd, Oxford, OX2 6GG UK; 2 Big Data Institute, University of Oxford, Li Ka Shing Centre for Health Information and Discovery, Old Road Campus, Oxford, OX3 7LF UK

**Keywords:** Malaria, Fever, RDT

## Abstract

**Background:**

Rapid diagnostic tests (RDTs) are increasingly becoming a paradigm for both clinical diagnosis of malaria infections and for estimating community parasite prevalence in household malaria indicator surveys in malaria-endemic countries. The antigens detected by RDTs are known to persist in the blood after treatment with anti-malarials, but reports on the duration of persistence (and the effect this has on RDT positivity) of these antigens post-treatment have been variable.

**Methods:**

In this review, published studies on the persistence of positivity of RDTs post-treatment are collated, and a bespoke Bayesian survival model is fit to estimate the number of days RDTs remain positive after treatment.

**Results:**

Half of RDTs that detect the antigen histidine-rich protein II (HRP2) are still positive 15 (5–32) days post-treatment, 13 days longer than RDTs that detect the antigen *Plasmodium* lactate dehydrogenase, and that 5% of HRP2 RDTs are still positive 36 (21–61) days after treatment. The duration of persistent positivity for combination RDTs that detect both antigens falls between that for HRP2- or pLDH-only RDTs, with half of RDTs remaining positive at 7 (2–20) days post-treatment. This study shows that children display persistent RDT positivity for longer after treatment than adults, and that persistent positivity is more common when an individual is treated with artemisinin combination therapy than when treated with other anti-malarials.

**Conclusions:**

RDTs remain positive for a highly variable amount of time after treatment with anti-malarials, and the duration of positivity is highly dependent on the type of RDT used for diagnosis. Additionally, age and treatment both impact the duration of persistence of RDT positivity. The results presented here suggest that caution should be taken when using RDT-derived diagnostic outcomes from cross-sectional data where individuals have had a recent history of anti-malarial treatment.

**Electronic supplementary material:**

The online version of this article (10.1186/s12936-018-2371-9) contains supplementary material, which is available to authorized users.

## Background

With resistance to first-line anti-malarials becoming increasingly widespread [1], malaria-endemic countries have made a shift towards parasite-based diagnosis of malaria infections in clinics rather than presumptive diagnosis of suspected cases in order to prevent over-prescription of anti-malarials and to curb anti-malarial resistance, owing in part to a change of World Health Organization (WHO) recommendation in 2010 [2]. Between 2010 and 2015, sales of rapid diagnostic tests (RDTs) from manufacturers worldwide tripled from 90 to 270 million, and in 2015 RDTs constituted 74% of diagnostic testing for suspected malaria cases [3]. RDTs have now joined microscopy as a mainstay of malaria diagnosis in household surveys, with a number of the most recent malaria indicator surveys (MIS) from the Demographic and Health Surveys (DHS) Programme using only RDTs, without microscopy [4, 5]. These data from national surveys have been used to produce spatiotemporal maps of malaria prevalence and mortality at a continental scale [6, 7] (after careful standardization across different diagnostic methods [8]), to estimate the proportion of febrile illness in African children that is attributable to malaria versus other causes [9], and to generate estimates of the effectiveness of health systems in malaria-endemic countries, amongst numerous other applications.

RDTs typically detect at least one of two antigens: histidine-rich protein II (HRP2), a protein occurring in the cytoplasm of *Plasmodium falciparum*; and *Plasmodium* lactate dehydrodgenase (pLDH), a glycolytic enzyme produced by live *Plasmodium* parasites of all human-infecting species. RDTs that detect pLDH either detect pan-pLDH (also commonly referred to as the pan-malarial antigen, or PMA), which is common to all human-infecting species, or species-specific pLDH. Most commonly, RDTs fall into two categories: detecting HRP2 only, or detecting both HRP2 and pan-pLDH. Of the 57 national surveys conducted by the DHS Programme released before the end of 2017, 21 surveys used HRP2-only RDTs, 33 used HRP2/pan-pLDH combination RDTs, 5 used HRP2/*Plasmodium vivax*-pLDH combination RDTs, and one used a pan-pLDH-only RDT (Pers. comm. from DHS Program, 2017). Within these surveys, 43.2% of individuals with negative RDT results at time of observation but who had sought treatment for their fever within the previous 2 weeks reported to have received anti-malarial medication at their treatment location. Some fraction of these individuals (who would typically be identified as non-malarial fever cases by end-users of the household survey dataset) may in fact have had a symptomatic malaria infection that was successfully treated in time to allow the malaria antigen concentration in their blood to reduce below the threshold for RDT detection at the survey interview. Amongst these (at-survey) RDT-negative patients who sought treatment and received an anti-malarial, 45.1% received an RDT diagnosis at their treatment location. Although the interviewers do not ask the result of the RDT administered at the treatment location, this figure is indicative of a high rate of RDT positivity at the time of treatment; a 2016 review of health worker compliance in clinics in malaria-endemic countries showed that only 1.5% of RDT-positive patients in clinics do not receive an anti-malarial, and that 19.1% of RDT-negative patients receive an anti-malarial (overtreatment) [10]. Understanding the quantitative impact of prior treatment on observed RDT status is an important step towards adding value from fever status records to the enumeration of malaria burden.

False negativity in RDT diagnosis, defined as the failure to register a positive result for patients having any non-zero parasite load, usually arises through parasite antigen concentrations within the blood being lower than the threshold of detection for RDTs, which accounts for approximately half of *P. falciparum* infections in malaria-endemic populations [11]. This threshold depends on the particular RDT and the expertise of the individual administering the diagnostic, but typically falls in the range of 5-15 parasites per μL [12]. Additionally, *P. falciparum* parasites with histidine-rich protein II (HRP2) deletions have been reported in more than 10 countries [3], which would lead to false negative results in RDTs that detect only the HRP2 antigen. Conversely, false positive results have been reported in patients expressing rheumatoid factor [13], and it remains a possibility that they could arise through non-specific binding of heterophilic antibodies [14]. The evidence collated in this review describes another route through which an individual without a current malaria infection could still present with a positive RDT: the persistence of malaria antigens after recent parasite clearance through anti-malarial medication. There is a temptation to view this outcome as a ‘false positive’ when imagining RDT as a proxy for microscopic detection, but when using RDTs for survey-based estimation of disease transmission intensity this is precisely the desired measure: evidence of recent parasite exposure. Understanding the duration of positivity after successful parasite clearance is also critical for accurate diagnosis of re-infection after a previous infection has been treated; if antigens from the first infection are still present in great enough quantities after treatment, an individual will still return a positive RDT after treatment, and may be misinterpreted as a re-infection, or, indeed, re-infections may be missed.

The amount of time necessary for RDTs to turn negative after treatment of an RDT-patent malaria infection is shaped by the different rates at which HRP2 and pLDH persist in the blood post-treatment; hence the type of RDT used is likely to be important. Additionally, the type of treatment the patient receives is critical to the speed and completeness of parasite clearance [15], and is, therefore, also likely to affect the time taken for the RDT to become negative. Parasite density at the time of anti-malarial medication has also been reported to correlate with increased duration of persistent positivity [16], so in malaria-endemic areas the age of the individual may influence the likelihood of persistent positivity given the link between acquired immunity, parasite density and age. Numerous studies that state that persistent antigenicity may pose a limitation to their findings typically reference one or two examples of persistent antigenicity in other publications [18–27]; however, the reported length of persistent antigenicity is highly variable amongst studies. In this analysis, data from published reports of persistent antigenicity are collated, systematically reviewing and synthesizing all information available on the length of time required for RDTs to turn negative after treatment of a malaria infection, and a Bayesian survival model is fit to estimate the length of time RDTs can be expected to remain positive after treatment, exploring how this duration is affected by the type of RDT used, the anti-malarial drugs administered, and the age of the individuals.

## Methods

### Systematic literature review

A systematic review of publications on the persistence of antigenaemia and positivity of RDTs after treatment was conducted using the search term “antigen persist RDT” on Google Scholar. Due to persistence of antigenicity typically being reported as a secondary result in publications evaluating RDT performance, indexing sites such as PubMed or Web of Science returned few results due to the inconsistency of terminology both in title and content for this type of publication (12 results on PubMed and 13 on Web of Science for the same search terms used in the Google Scholar review). Other search terms (“HRP2 persist”, “pLDH persist”, and “RDT persist positivity”) were attempted on PubMed and Web of Science and each yielded 5 or fewer results, all of which were non-relevant or identified in the final Google Scholar review. The Google Scholar search yielded more than 4100 results, sorted by relevance. The search continued until 10 consecutive pages of results yielded no more publications meeting the following inclusion criteria: they (i) treated patients for symptomatic malaria infections; and, (ii) followed up with RDT-based diagnosis over a number of days. The proportion of individuals who still tested positive via RDT was extracted for each day of follow-up and analysed as a time series. In addition to the proportion of positive tests, information on other factors that may affect RDT positivity in the follow up period was also extracted: (i) RDT type (antigens detected); (ii) treatment received (artemisinin-based combination therapy (ACT) or non-ACT); and, (iii) age range of patients (children 5 years of age or under, or adults over 14 years of age). There were two studies where the treatment regimen was unknown; 3 were conducted prior to 2001 so it was assumed that individuals received a non-ACT anti-malarial [28–30], and one conducted in 2011 so it was assumed that individuals received an ACT [23]. All studies meeting the inclusion criteria are documented in full in Additional file 1: Table S1.

### Bayesian survival model

The data was fit with a Bayesian survival model, where the time taken for an event to occur—in this case an individual becoming RDT-negative—is modelled. This time is determined by a hazard function $h\left( t \right)$ which represents the instantaneous probability of the event occurring given it has not already occurred. Therefore a high value of $h\left( t \right)$ corresponds to a high probability that an individual will become RDT-negative at time $t$, given they were not already RDT-negative. Given a hazard function $h\left( t \right)$, a survival curve $S\left( t \right)$ can be calculated which represents the probability of an individual still being RDT-positive at time.

It was assumed that there was a common baseline hazard function for all study groups, analogous to assuming the overall shape of a survival curve will be similar regardless of drug type, RDT type or age of the individual. Each of the variables (drug type, RDT type, age, year) then scaled the hazard function by a constant—a variable increasing the hazard function would corresponding to individuals being likely to become RDT-negative earlier.

A piecewise-constant baseline hazard function $h\left( t \right)$ was assumed across all study groups,

$$h\left( t \right) = c_{i}$$

for $t_{i} < t < t_{i + 1}$, with $c_{i}$ a non-negative constant and the time points, $t_{i}$, the times at which an RDT was carried out in any study. The group-specific hazard function for the *j*th study group, $h_{j} \left( t \right)$, was assumed to be this baseline hazard function modulated by the effect of covariates, $X_{j}$, and a study group-level random effect, $Z_{j}$, as follows:

$$h_{j} \left( t \right) = h\left( t \right) \times \exp \left( { - \left( {X_{j}^{T} \beta + Z_{j} } \right)} \right).$$

The covariates were all categorical and included: (i) age (child, unknown, adult); (ii) RDT type (HPR2 or pLDH, either alone or in combination with HRP2); (iii) drug type (ACT or non-ACT); and, (iv) year of study (i.e. the year the research was conducted rather than publication year; separated into 5 categories: 1990–1995, 1996–2000, 2001–2005, 2006–2010, and 2011–2013).

The cumulative hazard function $H_{j} \left( t \right)$ is given by the integral of the hazard function,

$$H_{j} \left( t \right) = \mathop \int \limits_{0}^{t} h_{j} \left( t \right)dt$$

and the survival curve, $S_{j} \left( t \right)$, is given by $S_{j} \left( t \right) = \exp \left( { - H_{j} \left( t \right)} \right)$. The value, $S_{j} \left( t \right)$, represents the probability of individual becoming RDT-negative at time, $t$, or later.

Finally, a probability of treatment failure, $p_{\text{fail}}$, was incorporated, and assumed dependent on all previous covariates except RDT type:

$$p_{\text{fail}} = {\text{logit}}^{ - 1} \left( {X^{\prime}_{j} \beta_{\text{fail}} } \right) = \frac{1}{{1 + { \exp }\left( { - X^{\prime}{_{j}^{T}} \beta_{\text{fail}} } \right) }}$$

where $X^{\prime}_{j}$ are the covariates other than RDT type and $\beta_{\text{fail}}$ are slopes learnt during the model fit.

Choosing suitable priors (see Additional file 2 for full details of priors and likelihoods), posterior distributions for $c$, $Z_{j}$ were approximated, and survival curves predicted by choosing the desired covariates and setting $Z_{j}$ to zero. Model fitting was performed in R using the TMB package for automatic differentiation [30].

As the model does not allow individuals to test positive after testing negative, any study groups where the percentage of still-positive individuals increased by more than 15% points between time points were removed. For study groups with smaller increases over time this data was included but with increases removed, as the general trend of the data would still be informative. Leave-one-out cross-validation was conducted to analyse predictive performance.

## Results

### Data

A total of 31 separate publications met the inclusion criteria, yielding 67 individual study groups, as some publications followed up multiple groups of individuals post-treatment. The studies included were conducted between 1994 and 2013. The number of individuals within each study group varied highly, ranging from 10 to 386 individuals (mean = 104.1, SD = 92.2). All studies incorporated only uncomplicated malaria cases, with the exception of one study group which incorporated both severe and uncomplicated malaria cases [31]. Twenty-eight of the study groups were confined to children 5 years of age or under, 3 to adults 14 years or older, 12 study groups were of mixed age, and the age range was unknown in 24 study groups. The anti-malarial received was recorded for all study groups; 44 of these study groups received an ACT while the remaining 23 study groups received a non-ACT anti-malarial. HRP2-only RDTs were used in 40 of the study groups, RDTs detecting pLDH-only were used in 21 study groups, and combination RDTs detecting both HRP2 and pLDH were used in 6 study groups. A total of 5 study groups were omitted entirely due to increases of over 15 percentage points in the proportion of positive individuals between consecutive measurements, and a further 9 study groups had outcomes from one or more days removed due to smaller increases. Full details of the extracted data, along with study references, can be found in Additional file 1: Table S1.

### Model fit

**Fig. 1:**
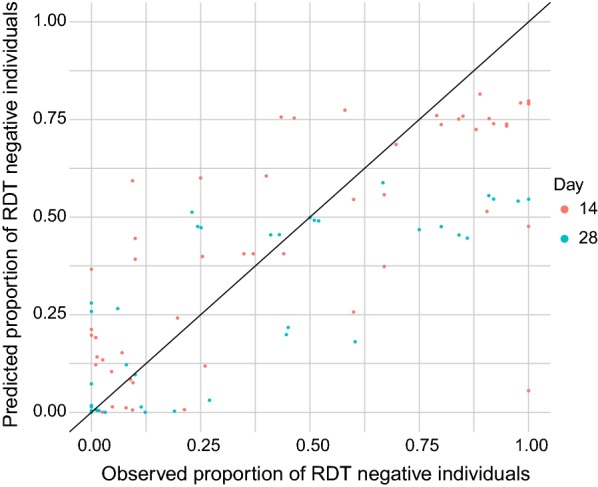
Observed and predicted proportion of RDT negative individuals at 14 and 28 days after treatment from leave-one-out cross-validation. The black line represents 1:1 for observations and predictions

Table 1 summarizes the estimated baseline hazard function. The value of the hazard function remains relatively constant until day 17 after treatment, suggesting a constant probability of reverting to RDT negative until that time. The value of the hazard function increases after 17 days post-treatment, indicative of an increasing probability of an individual who is still RDT-positive reverting to RDT-negative with each successive day post-treatment. The standard deviation of the baseline hazard function increases with time, likely due to the paucity of data more than 42 days post-treatment (Table 1). To test the predictive performance of the model leave-one-out cross-validation was performed. Each study group was removed iteratively from the response data and then compared the predicted and observed proportion of RDT-negative individuals in this group at 14 and 28 days. Correlations of 0.75 and 0.78 were found between observed and predicted values at 14 and 28 days, respectively (summarized in Fig. 1) showing good predictive performance.

**Table 1:** Estimated baseline hazard function for probability of RDTs reverting from positive to negative 0–63 days after treatment

Time interval (days)	Log hazard function (log *c*_i_)	
	Mean	Standard deviation
$0 \le t < 1$	− 2.14544	0.59800
$1 \le t < 2$	− 2.33424	0.59788
$2 \le t < 3$	− 2.22978	0.59812
$3 \le t < 4$	− 1.95530	0.59871
$4 \le t < 5$	− 2.06535	0.59897
$5 \le t < 6$	− 2.63433	0.59984
$6 \le t < 7$	− 2.88932	0.60210
$7 \le t < 10$	− 2.30567	0.61558
$10 \le t < 14$	− 2.11019	0.60812
$14 \le t < 17$	− 2.00743	0.62575
$17 \le t < 21$	− 1.82409	0.61273
$21 \le t < 28$	− 1.64152	0.60006
$28 \le t < 35$	− 0.95173	0.59972
$35 \le t < 42$	− 0.76698	0.60509
$42 \le t < 49$	− 0.40862	0.66399
$49 \le t < 56$	− 0.32584	0.73220
$56 \le t \le 63$	0.06540	0.89587

Table 2 summarizes the model-estimated coefficients for factors affecting duration of persistent positivity. Higher values correspond to a lower hazard function and thus increased probability of a longer duration of persistent RDT positivity. In addition to the primary factors (age, RDT type, anti-malarial medication type) described in more detail below, the effect of year of study on the hazard function was investigated. A small effect was observed that older studies (pre-2000) and those conducted between 2006 and 2010 show shorter durations of persistent positivity than those conducted between 2001 and 2005 and those conducted between 2011 and 2013. The coefficients for all variables affecting the likelihood of treatment failure are outlined in Table 3.

**Table 2:** Estimated coefficients for variables affecting duration of persistent positivity

Variable	*β*	
	Mean	Standard deviation
Age		
Child	0.47259	0.34271
Unknown	− 0.17405	0.34302
Adult	− 0.31372	0.35144
RDT type		
HRP2	0.92519	0.33919
pLDH	− 1.04777	0.33905
Combination	0.10741	0.34083
Drug type		
ACT	0.25571	0.41472
Non-ACT	− 0.27088	0.41472
Year of study		
1990–1995	− 0.32517	0.29396
1996–2000	− 0.0111	0.26762
2001–2005	0.36172	0.26733
2006–2010	− 0.08271	0.26692
2011–2013	0.04209	0.26782

Higher coefficient values correspond to a lower hazard function and thus increased probability of a longer duration of persistent RDT positivity

**Table 3:** Estimated coefficients for variables affecting probability of treatment failure

Variable	*β*_fail_	
	Mean	Standard deviation
Age		
Child	− 3.46444	2.57147
Unknown	− 0.07017	2.15373
Adult	− 0.39833	2.2029
Drug type		
ACT	− 2.00807	2.38318
Non-ACT	− 1.42487	2.38322
Year of study		
1990–1995	− 0.00101	1.66774
1996–2000	− 0.90702	1.61296
2001–2005	− 0.9747	1.61356
2006–2010	− 0.54851	1.6232
2011–2015	− 2.5017	3.76325

Higher coefficient values correspond to a higher probability of treatment failure

### Overall survival estimate

**Fig. 2:**
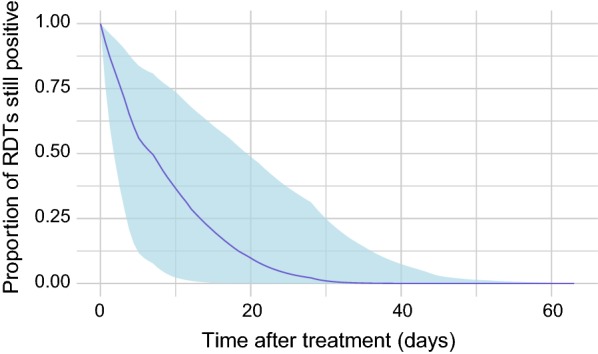
Fitted relationship between proportion of RDTs still positive within a given sample of individuals, and length of time (number of days) after treatment was first administered. All study groups were used in this fitted relationship. The posterior distribution median (blue line) and 95% credible intervals (light blue shaded area) is displayed

Overall, there was substantial variability in the proportion of RDTs that remained positive 1–63 days after treatment. In some study groups, the RDTs of all patients were negative 2–3 days after treatment, but in one study group some individuals still returned a positive RDT 56 days after treatment. Clustering in measurement days at 7, 14, 21, and 28 days after treatment was observed, with sparsity in measurements in the intervening days. Figure 2 shows the fitted estimate of persistence of RDT positivity 0–63 days after treatment for all study groups, overlain with the proportion of individuals still positive within each study group at each time point. The fitted Bayesian survival model estimates that, in any given cross-sectional population, 50% of treated individuals will present a negative RDT at 7 days (2–20 days, 95% CI) post-treatment, and that 95% of individuals will present a negative RDT by 24 days (8–43 days, 95% CI) post-treatment.

### Likelihood of persistent positivity by RDT type

Study groups were separated by the type of RDT used in the analysis; HRP2-only RDTs were used in 40 study groups. 21 study groups were analysed pLDH-only RDTs (including pan-*Plasmodium* pLDH and *P. falciparum* pLDH variants). 6 study groups included combination RDTs which tested for both HRP2 and pan-*Plasmodium*-pLDH. Two study groups using HRP2 RDTs and 3 study groups using combination RDTs were removed prior to analysis due to increases in proportion of positive individuals over time. RDTs from a total of 7 different manufacturers were used across the study groups (Additional file 1: Table S1). For 3 of the studies, a single combination HRP2/pan-pLDH RDT was used, but instead of a binary positive/negative result, the result for each individual band was given [32–34]. These studies are included as two separate study groups in both the HRP2-only group and the pLDH RDTs group, with the result for the relevant band included in each group. The binary results for all other combination tests where individual band positivity was unknown were included in the combination RDTs group.

**Fig. 3:**
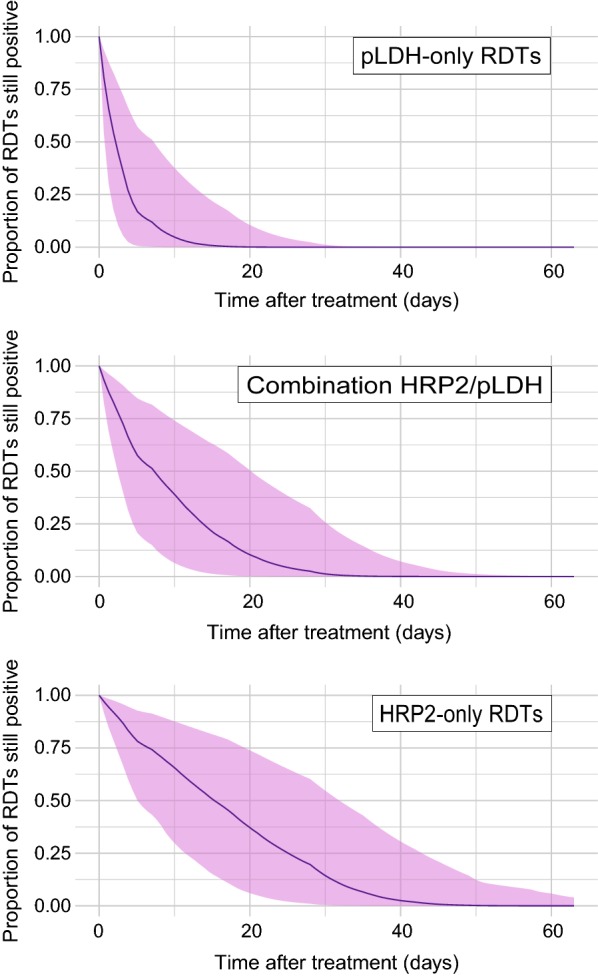
Fitted relationship between proportion of RDTs still positive within a given sample of individuals, and length of time (number of days) after treatment was first administered, separated by the type of RDT used to monitor patients during the follow-up period. The fitted relationship for study groups monitored with RDTs that detect pLDH only (*N* study groups = 21) is shown in the top panel (with shaded 95% credible intervals), and the fitted relationship for study groups monitored with RDTs that detect pLDH in combination with HRP2 (*N* study groups = 6) is in the middle panel (with shaded 95% credible intervals), and the fitted relationship for study groups monitored with RDTs that detect HRP2 only (*N* study groups = 40) is in the bottom panel (with shaded 95% credible intervals)

In Fig. 3, the fitted survival model estimate for each of the 3 RDT types is shown. Table 2 shows the model-estimated coefficients for the effect of RDT type, with HRP2 RDTs showing a significantly higher probability of experiencing a longer duration of persistent positivity than other RDTs. The model predicts that, within a population monitored using HRP2 RDTs, 50% of treated individuals would present with a negative RDT by 15 days (5–32 days, 95% CI) after treatment, and 95% of individuals would present with a negative RDT by 36 days (21–61 days, 95% CI) after treatment. For RDTs detecting pLDH only, 50% of individuals would present with a negative RDT by 2 days (1–7 days, 95% CI) after treatment, and 95% of individuals present with a negative 10 days (3–24 days, 95% CI) after treatment. Duration of persistent positivity for combination RDTs that detect both HRP2 and pLDH falls between HRP2 and pLDH RDTs, with 50% of RDTs presenting a negative result by 7 days (2–20 days, 95% CI) and 95% of RDTs presenting a negative result by 24 days (11–43 days, 95% CI).

### Likelihood of persistent positivity by type of therapy received

Type of treatment received on day 0 of the analysis was known for all study groups. Treatment regimens were grouped based on whether or not they were considered ACT (i.e., an artemisinin derivative coupled with a partner anti-malarial drug). Non-ACT included artemisinin monotherapy, chloroquine, quinine, primaquine, sulfadoxine–pyrimethamine, and mefloquine (full details of all treatment regimens can be found in Additional file 1: Table S1). In total, 44 study groups received ACT, and 23 study groups received non-ACT treatment. Three study groups receiving non-ACT and two study groups receiving ACT were removed prior to analysis due to increases in proportion of positive individuals over time.

**Fig. 4:**
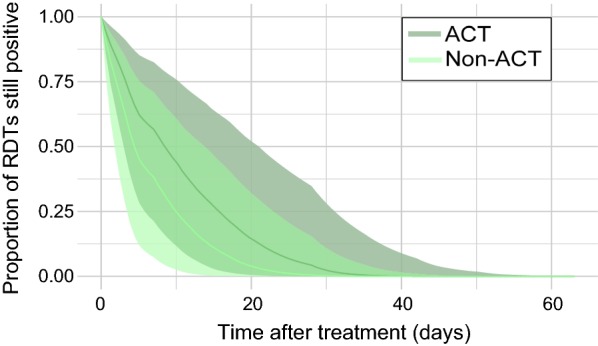
Fitted relationship between proportion of RDTs still positive within a given sample of individuals, and length of time (number of days) after treatment was first administered, separated by the type of anti-malarial medication administered to patients on day 0 of the analysis. The dark green line (with shaded 95% credible intervals) shows the fitted relationship for individuals who received an ACT on day 0 (*N* study groups = 44), and the fitted relationship for individuals who received a non-ACT anti-malarial medication (either artemisinin monotherapy, chloroquine, quinine, primaquine, sulfadoxine-pyrimethamine, and mefloquine—full details in Additional file 1: Table S1; *N* study groups=23) is shown by the light green line (with shaded 95% credible intervals)

Table 2 shows the model-estimated coefficients for the effect of treatment, with individuals who received an ACT showing a higher probability of experiencing a longer duration of persistent positivity than those who received a non-ACT, although the magnitude of the difference between the coefficients is less pronounced than the effect of RDT type, and the credible intervals for these estimates include zero, indicating a higher level of uncertainty. The fitted relationship between persistent RDT positivity and length of time since treatment, separated by the type of therapy received, is shown in Fig. 4. The model predicts that 50% of patients who received an ACT would test negative via RDT by day 8 (3–21, 95% CI), compared to day 4 (2–14, 95% CI) for patients that received a non-ACT anti-malarial. It is predicted that 95% of patients that received an ACT would test negative via RDT by day 27 (13–43, compared to day 19 (8–34, 95% CI) for patients that received a non-ACT anti-malarial.

### Combined effect of RDT type and therapy received

**Fig. 5:**
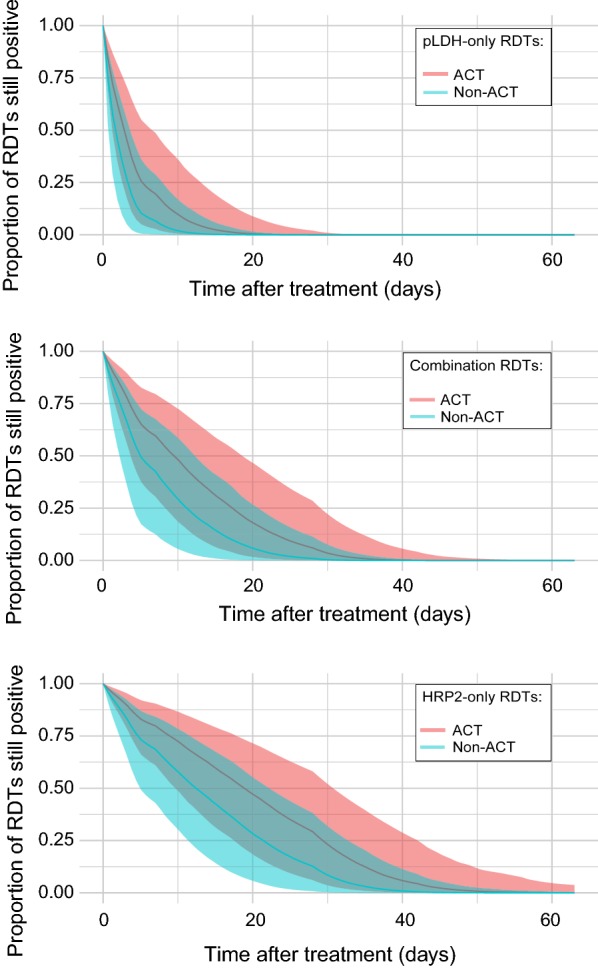
Both panels show the fitted relationship between proportion of RDTs still positive within a given sample of individuals, and length of time (number of days) after treatment was first administered, separated by the type of anti-malarial medication administered to patients on day 0 of the analysis. Top panel: fitted relationship by type of anti-malarial medication amongst study groups tested with a pLDH-only RDT. Middle panel: fitted relationship type of anti-malarial medication amongst study groups tested with a HRP2/pLDH combination RDT. Bottom panel: fitted relationship by type of anti-malarial medication amongst study groups tested with a HRP2-only RDT. In each panel, the fitted relationship for individuals who received an ACT is shown by the pink line (with shaded 95% credible intervals), and the fitted relationship for individuals who received a non-ACT is shown by the blue line (with shaded 95% credible intervals)

Figure 5 shows the effect of type of anti-malarial therapy received on the fitted relationship when separated by RDT type. For both categories of anti-malarial, persistent positivity is predicted for a longer duration when using HRP2 RDTs than when using pLDH-only or HRP2/pLDH combination RDTs. When using HRP2-only RDTs during follow-up, the model predicts that 50% of patients would present with a negative test by day 19 (10–31, 95% CI) when given an ACT on day 0, or by day 13 (5–22, 95% CI) when given a non-ACT at day 0; and the model predicts that, using a HRP2 RDT, 95% patients would present with a negative test by day 41 (29–59, 95% CI) when given an ACT on day 0, or by day 32 (21–46, 95% CI) when given a non-ACT at day 0. When using pLDH-only RDTs during follow-up, the model predicts that 50% of patients would present with a negative test by day 3 (1–6, 95% CI) when given an ACT on day 0, or by day 2 (1–4, 95% CI) when given a non-ACT at day 0; and, using pLDH-only RDTs, 95% patients would present with a negative test by day 13 (5-23, 95% CI) when given an ACT on day 0, or by day 8 (3–16, 95% CI) when given a non-ACT at day 0. For HRP2/pLDH combination RDTs, 50% of patients would present with a negative test by day 10 (4–18, 95% CI) when given an ACT on day 0, or by day 5 (2–12, 95% CI) when given a non-ACT at day 0; and, 95% patients would present with a negative test by day 29 (16–41, 95% CI) when given an ACT on day 0, or by day 21 (10–32 95% CI) when given a non-ACT at day 0.

### Likelihood of persistent positivity by age of patient

**Fig. 6:**
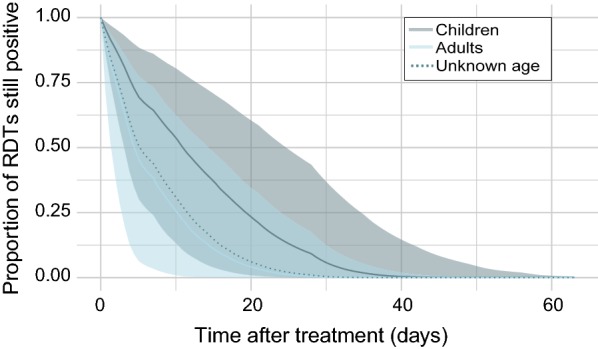
Fitted relationship between proportion of RDTs still positive within a given sample of individuals, and length of time (number of days) after treatment was first administered, separated by age. Children 5 years of age or under (*N* study groups = 28) are represented by the dark blue line and shaded 95% credible intervals; adults 14 years of age or older (*N* study groups = 3) are represented by the light blue line and shaded 95% credible intervals. Mixed and unknown ages (*N* study groups = 36) are represented by the dotted blue line (credible intervals are not shown)

The age of sampled individuals was known for 41 of the 67 study groups. Of these, 28 of the study groups were confined to children 5 years of age or under, 3 to adults 14 years or older and 12 study groups were of mixed age (Additional file 1: Table S1). One study group concerning children 5 years of age or under and one study group concerning adults were removed prior to analysis due to increases in proportion of positive individuals over time. In Table 2, the model-estimated coefficients for the effect of age of individuals can be found. The model predicts that children are more likely to experience longer durations of persistent positivity than adults, although again the magnitude of the difference between the coefficients is less pronounced than the effect of RDT type, and again, the credible intervals for the estimate for adults are wide, indicating a high level of uncertainty. In Fig. 6, the fitted relationship between persistent positivity and days since treatment is shown, confined separately to only children 5 years of age or under, and only adults 14 years of age or older. Children were estimated to have a slightly longer duration of persistent positivity than adults; it is estimated that 50% of children would present a negative RDT by day 11 (3–25, 95% CI), whereas 50% of adults would present a negative RDT by day 4 (1–15, 95% CI). The model estimates that 95% of children would present a negative RDT by day 31 (14–49, 95% CI), and 95% of adults would present a negative RDT by day 19 (6–36, 95% CI).

## Discussion

In this study, published data on persistence of positive RDT results after anti-malarial treatment is collated. Amongst the study groups, the proportion of individuals who still test positive at increasing numbers of days after treatment is highly variable (Fig. 2) but shows more discernible trends when grouped by the type of RDT used to detect the infection (Fig. 3) and the treatment that the individuals receive at day 0 (Fig. 4). The Bayesian survival model estimates presented here can be used as a tool to estimate the proportion of individuals within a given study group that would still present a positive RDT for 2 months after anti-malarial treatment, and allow further distinction depending on: (i) RDT type used for analysis; (ii) type of treatment administered at day 0; and, (iii) age of patients. On average, the model predicts that half of RDTs will be negative 7 days after treatment has been received.

### RDT type

The finding that HRP2 RDTs show persistent positive results after treatment for longer than combination or pLDH RDTs (Figs. 4, 5) corroborates direct comparisons between HRP2 and pLDH RDTs amongst the same patients [17, 35, 36], owing to the slower degradation of HRP2 compared to pLDH after parasite clearance. The duration of persistent positivity for combination RDTs that detect both HRP2 and pLDH falls between the durations for RDTs that detect only one antigen; this suggests that the positivity of the HRP2 line on the combination test is behaving similarly to HRP2-only tests and that studies are often reporting an RDT to be positive if the HRP2 line indicates a positive result, even if the pLDH line does not indicate a positive.

Despite most study groups using HRP2 RDTs, more variability was observed in the length of time HRP2 RDTs returned positive results than for other RDTs, as demonstrated by the width of the credible intervals in Fig. 3. Mayxay et al. [31] measured the persistence of detectable levels of HRP2 post-treatment and found a clear relationship between longer persistence and higher levels of initial blood parasite density. In the dataset collated for this study, parasite density ranges were known for 22 of the 67 study groups (Additional file 1: Table S1) but the ranges of parasite density are wide and irregular, making analysis by initial parasite density unreliable and challenging. Future extensions of studies on persistent antigenicity would benefit from recording parasite density, or even the severity of a patient’s symptoms, as fever severity is linked to increased parasite density [37]. Although pLDH degrades faster than HRP2, both RDTs return positive results for a long time post-treatment; the model estimates that some HRP2 RDTs will still be positive more than 36 days after treatment, and RDTs detecting pLDH for more than 10 days after treatment. Positive pLDH RDTs could result from the persistence of malaria gametocytes, as they are unaffected by some anti-malarials but are known to produce pLDH in mature gametocytes (but not HRP2, which is only produced by immature gametocytes) [38, 39]. Persistent positivity of combination RDTs could be due to ambiguity in the reporting of a “positive” result with HRP2/pLDH combination RDTs. For example, if a recently treated individual returns a positive HRP2 band and a negative pan- or *Pf*-pLDH band on the same RDT, whether or not this result would be interpreted as a true or false positive would depend on the training of the clinician and the perceived reliability of the test. Differences in positivity by HRP2 band and pLDH band were not reported for a number of the studies that included combination RDTs. In a number of publications, a test was considered “positive” if either band was clearly visible. Clear division in the positivity of each band would help characterize the different reactivities of HRP2 and pLDH bands to blood after anti-malarial treatment, as combination RDTs with only a HRP2 band visible (caused by the prolonged degradation of HRP2) may simply be recorded as “positive”, thus skewing the results for combination RDTs in favour of longer positivity duration. This method of defining malaria “positivity” also presents a problem from a converse perspective: if a treated *P. falciparum* infection generated a negative HRP2 band and a positive pan-pLDH band (due, perhaps, to circulating mature gametocytes not cleared by anti-malarial medication and producing pLDH), then the RDT may be misreported as a malaria infection by a species other than *P. falciparum* [39]. This type of false negativity of HRP2 bands could also arise should the parasite be carrying a HRP2 deletion, which while currently not thought to be widespread, has been reported in 5 countries in sub-Saharan Africa [40] and 10 countries worldwide [3].

### Type of anti-malarial therapy received

In Fig. 4, the proportion of individuals still positive at increasing numbers of days shows that the study groups who received ACT are highly variable in the rate at which RDTs become negative, compared to the groups who received non-ACT. When broken down by the type of RDT used to measure persistent positivity, individuals who took ACT remained persistently positive for longer than individuals who took non-ACT anti-malarials. The reasons for this are unclear, and may seem counterintuitive. A potential explanation could relate to individuals who received ACT being tested in later years due to introduction of ACT as the first-line anti-malarial in 2006 (the publications included here have a mean study date of 2007 for those who receive ACT), compared to non-ACT anti-malarials (mean study date of 2001 for those who receive non-ACT), which may not have been fully captured in the random study effect. Over the past 2 decades, RDTs have increased greatly in sensitivity, as reported by the World Health Organization’s Malaria Rapid Diagnostic Test Performance, which has undergone 7 rounds of product testing of RDTs, with the latest round conducted in 2015–2016 [41].

Studies comparing persistent antigenicity with separate groups testing ACT and non-ACT anti-malarials find variable differences in HRP2-only RDT positivity between trial groups: including a study by Houze et al. comparing ACT and sulfadoxine-pyrimethamine which found no difference [35], and a study by Tjitra et al. comparing sulfadoxine-pyrimethamine and artesunate with sulfadoxine-pyrimethamine (AS-SP) which found that AS-SP had approximately one-third fewer persistently positive RDTs in the first 7 days after treatment, and then roughly equivalent rates [42].

Due to the small number of study groups tested with non-ACT anti-malarials, and the variety of anti-malarials used within this group, non-ACT anti-malarials were not disaggregated into individual drugs. As the particular drug used tends to be a known variable in both clinical settings and for prevalence surveys, the analysis presented here could be improved by repeating for individual non-ACT drugs (and even different combinations within the ACT category) if more data from non-ACT study groups were available.

### Relationship between patient age and persistent RDT positivity

The results presented here show that children are more likely to remain positive for a longer time after anti-malarial treatment than adults, although the model fits have significant overlap in their 95% credible intervals. This significant overlap is in part due to the small sample size of study groups formed of only adults; age range was unknown for 24 of the study groups, and the remainder included individuals of all ages, and disaggregating observations by the age of individuals was not possible. Children are less likely to have developed acquired immunity to malaria than adults [43]. Acquired immunity leads to lower parasite densities [44], which has been reported to lead to a shorter duration of persistent RDT positivity after treatment [16]. The finding is also strengthened by the model estimates for unknown or mixed age individuals (represented by the dotted line in Fig. 6), which falls between the estimates for children and adults.

### Applications and limitations of findings

Many of the publications included as data points in this analysis report persistent antigenicity as a secondary finding of other analyses, such as evaluations of new RDTs [45–47] or testing RDTs in a new setting, such as introducing RDTs into clinics in malaria-endemic areas where previously only microscopy or presumptive diagnosis were available [18, 48–51]. A pitfall of this opportunistic data gathering approach is that the studies do not all follow the same protocol, and are conducted in a variety of settings. The findings of this review could be built on by a study designed with persistent antigenicity as the main subject of analysis, following rigorous protocol and testing the same subjects with a variety of RDTs (as some of the studies included here show persistent antigenicity as a main goal, but use only one type of RDT [37, 52–55]), although it would be unethical to provide RDT-positive subjects with anything other than first-line anti-malarial treatment. Additionally, many of the studies did not confirm (using microscopy or PCR) the species of *Plasmodium* infecting study groups. Many of the studies were conducted in sub-Saharan Africa and thus were likely to be reporting *P. falciparum* infections only [55] and most study groups used HRP2 RDTs (Additional file 1: Table S1), thus meaning only *P. falciparum* infections would be detected, but the possibility remains that some of the infections in study groups using pLDH or combination RDTs reported other species of *Plasmodium.* Antigen concentrations may differ between different *Plasmodium* species infections, so further analyses of persistent positivity would benefit from separating analysis by species.

A number of studies included here did not control for treatment failure or re-infection over the study period. The probability of treatment failure was controlled within the model, using known treatment failure rates as collated by the WHO [56]; however there is a small possibility that some of the still-positive RDTs seen in later days of the study period could be due to re-infection rather than persistent antigenicity. Re-infection is somewhat unlikely given the duration of the follow-up period in most of the studies included here, in addition to the prophylactic effect of the drugs administered at day 0, and the incubation time of malaria (9–15 days for *P. falciparum* and 12–17 days for *P. vivax* [57]).

Some of the studies included in this analysis described intensity of the indicator bands on the RDTs, but not a sufficient number, and for those that did include this information it was not sufficiently consistent for direct comparison. Band intensity is an indicator of parasite density (and subsequently antigen concentration) [58]. Further analysis of the band intensity change during the follow-up period after treatment may allow differentiation between persistent antigenicity and recrudescence or reinfection.

The WHO and the Foundation for Innovative New Diagnostics test the performance of RDTs systematically in the Product Testing Programme, using panel detection scores (PDS) as a guide to test sensitivity and specificity, and currently recommend that only RDTs with a panel detection score of over 75% are procured by national malaria control programmes (NMCP) [42] (although over the period 2011–2014, many NMCPs were not completely adherent to this criteria, especially amongst RDTs supplied to the private sector [59]). A number of the RDTs included in this analysis fall below the PDS threshold, although this threshold has moved over time, and many RDTs that currently fall below the PDS threshold were acceptable at the time the study was conducted. The RDTs included here show some intra-specific variation amongst RDTs that detect the same antigen(s), and future analyses would benefit from separating RDTs by the model rather than just the antigens detected, which was not possible in this analysis due to lack of reporting of RDT model number in the included studies. End-users of these results should take note of the sensitivity of the RDT used in their analysis relative to the sensitivities of the RDTs included in this study; more sensitive RDTs are likely to display longer durations of persistent positivity than the results presented here would indicate.

The findings presented here can be used to assess the reliability of positive RDTs in situations where it is known that the patient was treated for a patent malaria infection in the recent past. From the clinical perspective, the data presented here may be used to assess the likelihood of a non-malarial febrile illness as the underlying cause of an individual’s fever, should that individual present with a positive RDT but has also been known to have received anti-malarial treatment in the recent past. A recent study estimates that 72% of RDT-positive fevers in sub-Saharan African children are actually due to non-malarial causes [9], suggesting potential for improvements to the management of paediatric non-malarial fevers. For individuals with a current fever and a recent history of anti-malarial treatment, a positive RDT after successful anti-malarial treatment has potential as an indicator for non-malarial febrile illness (for which routine diagnostic tests are often unavailable) as the fever’s underlying cause is likely to be a non-malarial fever in this scenario, especially if the anti-malarial treatment received was an ACT. From the perspective of an analyst using household survey data on malaria prevalence, these results show that caution must be taken when a positive RDT from an individual who has also received recent anti-malarial treatment is observed. The Bayesian survival model estimates presented here can be used to give an estimate of the likelihood of persistent positivity, given the RDT used in the household survey, the treatment the individual has received, the age of the individual, and the number of days since the completion of treatment.

## Conclusion

This study shows that RDTs remain positive for a highly variable amount of time after treatment with anti-malarials. When grouped by antigen, RDTs that detect HRP2 only are found to show longer durations of persistent positivity than RDTs that detect pLDH; this difference is the most distinct effect from any of the factors investigated in this analysis. The model estimates that individuals receiving ACT are more likely to experience a longer duration of persistent positivity than individuals who receive non-ACT anti-malarials; this may, however, be an artefact of the coincident trend towards use of ACT as front-line anti-malarials and ever-increasing RDT sensitivities not fully captured by the random effects structure used in this analysis. Additionally, the results show that children are more likely to experience a longer duration of persistent positivity than adults. Epidemiologists combining RDT-derived household survey data on malaria prevalence with fever status reports and clinicians diagnosing malaria infections alike should take consideration of previous anti-malarial diagnosis and treatment, given the high likelihood of persistent positivity in the weeks succeeding anti-malarial treatment.

## Additional files

**Additional file 1: Table S1.** Details of the publications used in analysis.

**Additional file 2.** Bayesian survival model priors and likelihoods.
