# Supplementary material for: How long do rapid diagnostic tests remain positive after anti-malarial treatment?
Source: Malar J. 2018 Jun 8;17:228. doi: 10.1186/s12936-018-2371-9 (PMC5994115; doi:10.1186/s12936-018-2371-9)
Supplement: Supplementary file 1 — Additional file 1: Table S1. Details of the publications used in analysis. [file 12936_2018_2371_MOESM1_ESM.pdf]

Additional File 1. Details of the publications used in analysis.

																		Days after treatment - percentage of individuals still RDT positive																	
**ID**	**Author**	**Country**	**Month Start**	**Year Start**	**Month End**	**Year End**	**RDT brand**	**RDT model/lot number**	**RDT type †**	**Lines on same test ^**	**LowAge**	**UpAge**	**Selection of participants**	**Treatment type**	**Drug variety**	**Parasite density (per µL)**	**N**	**1**	**2**	**3**	**4**	**5**	**6**	**7**	**10**	**14**	**17**	**21**	**28**	**35**	**42**	**49**	**56**	**63**	**Points omitted? *****
1	Kyabinze	Uganda	10	2006	9	2007	ICT & Paracheck	Unknown	HRP2	No	0.5	4	Febrile	National treatment guidelines	ACT	?	162	-	-	100	-	-	-	97	-	88	-	63	41	18	9	3	1	0	None
2	Mbabazi	Uganda	11	2011	1	2012	Carestart	D21MO (September 2013)	HRP2	No	0	1	Febrile	ACT (artemether-lumefantrine or dihydroartemisinin–piperaquine)	ACT	?	131	-	94	-	-	-	-	91	-	79	-	68	51	-	-	-	-	-	None
3	Mbabazi	Uganda	11	2011	1	2012	Carestart	H11MY (July 2013)	pLDH (Pf)	No	0	1	Febrile	ACT (artemether-lumefantrine or dihydroartemisinin–piperaquine)	ACT	?	131	-	5	-	-	-	-	1	-	0	-	0	0	-	-	-	-	-	None
4	Kattenburg	Burkina Faso	11	2010	12	2010	SD Bioline	Unknown	HRP2	No	?	?	Pregnant women antenatal care	ACT (artesunate-amodiaquine, arthemeter-lumefantrine or artesunate-mefloquine)	ACT	?	32	78	66	59	-	-	-	41	-	25	-	9	6	-	-	-	-	-	None
5	Swarthout	DRC	3	2004	6	2004	Paracheck	Unknown	HRP2	No	0.5	4	Febrile	ACT (artesunate-amodiaquine or artesunate + sulphadoxine-pyrimethamine)	ACT	50 to 200,000	30	-	-	-	-	-	-	100	-	100	-	100	100	77.3	-	-	-	-	None
6	Swarthout	DRC	3	2004	6	2004	Paracheck	Unknown	HRP2	No	0.5	4	Febrile	ACT (artesunate-amodiaquine or artesunate + sulphadoxine-pyrimethamine)	ACT	20 to 50,000	42	-	-	-	-	-	-	100	-	100	-	97.6	97.6	79.2	-	-	-	-	None
7	Swarthout	DRC	3	2004	6	2004	Paracheck	Unknown	HRP2	No	0.5	4	Febrile	ACT (artesunate-amodiaquine or artesunate + sulphadoxine-pyrimethamine)	ACT	10 to 20,000	22	-	-	-	-	-	-	100	-	100	-	100	90.9	71.4	-	-	-	-	None
8	Swarthout	DRC	3	2004	6	2004	Paracheck	Unknown	HRP2	No	0.5	4	Febrile	ACT (artesunate-amodiaquine or artesunate + sulphadoxine-pyrimethamine)	ACT	2 to 10,000	18	-	-	-	-	-	-	94.4	-	88.9	-	72.2	66.7	50	-	-	-	-	None
9	Swarthout	DRC	3	2004	6	2004	Paracheck	Unknown	HRP2	No	0.5	4	Febrile	ACT (artesunate-amodiaquine or artesunate + sulphadoxine-pyrimethamine)	ACT	2 to 200,000	112	-	-	-	-	-	-	99.1	-	98.2	-	94.6	92	73.5	-	-	-	-	None
10	Ly	Senegal	10	2008	1	2009	Paracheck	Unknown	HRP2	No	0**	99**	Febrile	ACT (combination unknown)	ACT	?	10	-	-	-	60	-	-	40	-	-	-	-	-	-	-	-	-	-	None
11	Houze	Benin	4	2007	11	2007	Immunoquick	Unknown	HRP2	No	0.5	4	Febrile	ACT (artesunate-amodiaquine)	ACT	?	80	-	-	80.9	-	-	-	72.8	-	43.4	-	26.4	24.3	2.6	2.6	-	-	-	None
12	Houze	Benin	4	2007	11	2007	Optimal	Unknown	pLDH (Pan & Pf)	No	0.5	4	Febrile	ACT (artesunate-amodiaquine)	ACT	?	80	-	-	12.3	-	-	-	3.8	-	2.6	-	3	1.7	2.6	0	-	-	-	Some
13	Houze	Benin	4	2007	11	2007	Immunoquick	Unknown	HRP2	No	0.5	4	Febrile	Sulfadoxine-pyrimethamine	Non-ACT	?	44	-	-	76.2	-	-	-	60.9	-	35.7	-	17.9	50	19.6	0	-	-	-	All
14	Houze	Benin	4	2007	11	2007	Optimal	Unknown	pLDH (Pan & Pf)	No	0.5	4	Febrile	Sulfadoxine-pyrimethamine	Non-ACT	?	44	-	-	46.8	-	-	-	38.7	-	21.3	-	17.9	12.3	0	0	-	-	-	None
15	Houze	Benin	4	2007	11	2007	Immunoquick	Unknown	HRP2	No	0.5	4	Febrile	ACT (artemether-lumefantrine)	ACT	?	81	-	-	86	-	-	-	69.8	-	46.4	-	30.2	25.1	4.7	7.2	-	-	-	Some
16	Houze	Benin	4	2007	11	2007	Optimal	Unknown	pLDH (Pan & Pf)	No	0.5	4	Febrile	ACT (artemether-lumefantrine)	ACT	?	81	-	-	5.1	-	-	-	2.6	-	1.3	-	1.3	1.3	2.6	0	-	-	-	Some
17	Mohammed	Ethiopia	10	2008	1	2009	Paracheck	Unknown	HRP2	No	1	20	Febrile	ACT (artemether-lumefantrine)	ACT	?	80	-	-	-	-	-	-	98.7	-	60	-	48.1	44.6	-	-	-	-	-	None
18	Aydin-Schmidt	Tanzania	6 (1) 7 (2)*	2009 (1) 2010 (2)*	9 (1) 10 (2)*	2009 (1) 2010 (2)*	ParaHit	Unknown	HRP2	No	0.5	5	Febrile	ACT (artemether-lumefantrine)	ACT	2000 to 250,000	43	100	100	100	-	-	-	97	-	80	-	64	43	10	7	-	-	-	None
19	Aydin-Schmidt	Tanzania	6 (1) 7 (2)*	2009 (1) 2010 (2)*	9 (1) 10 (2)*	2009 (1) 2010 (2)*	CareStart	G0151	pLDH (Pf)	No	0.5	5	Febrile	ACT (artemether-lumefantrine)	ACT	2000 to 250,000	43	100	77	60	-	-	-	22	-	1	-	0	0	0	0	-	-	-	None
20	Huong	Vietnam	7	2000	9	2000	ICT	Unknown	HRP2/pLDH (Pan)	No	4	60	Febrile	ACT (artesunate-mefloquine)	ACT	?	87	-	-	-	-	-	-	50	-	0	-	0	0	-	-	-	-	-	None
21	Huong	Vietnam	7	2000	9	2000	Paracheck	Unknown	HRP2	No	4	60	Febrile	ACT (artesunate-mefloquine)	ACT	?	87	-	-	-	-	-	-	80	-	40	-	20	0	-	-	-	-	-	None
22	Tjitra	Indonesia	3	1999	4	1999	ICT	Unknown	HRP2	No	0**	99**	Febrile	Chloroquine	Non-ACT	?	66	66	78	83	-	-	-	59	-	37	-	-	10	-	-	-	-	-	Some
23	Tjitra	Indonesia	3	1999	4	1999	ICT	Unknown	HRP2/pLDH (Pan)	No	0**	99**	Febrile	Chloroquine	Non-ACT	?	66	33	55	55	-	-	-	50	-	37	-	-	5	-	-	-	-	-	All
24	Tjitra	Indonesia	3	1999	4	1999	ICT	Unknown	HRP2	No	0**	99**	Febrile	Sulfadoxine-pyrimethamine	Non-ACT	?	36	50	43	39	-	-	-	30	-	10	-	-	8	-	-	-	-	-	None
25	Tjitra	Indonesia	3	1999	4	1999	ICT	Unknown	HRP2/pLDH (Pan)	No	0**	99**	Febrile	Sulfadoxine-pyrimethamine	Non-ACT	?	36	40	55	50	-	-	-	42	-	19	-	-	0	-	-	-	-	-	All
26	Tjitra	Indonesia	3	1999	4	1999	ICT	Unknown	HRP2	No	0**	99**	Febrile	ACT (artesunate + sulphadoxine-pyrimethamine)	ACT	?	15	86	86	91	-	-	-	91	-	67	-	-	45	-	-	-	-	-	Some
27	Tjitra	Indonesia	3	1999	4	1999	ICT	Unknown	HRP2/pLDH (Pan)	No	0**	99**	Febrile	ACT (artesunate + sulphadoxine-pyrimethamine)	ACT	?	15	24	14	8	-	-	-	0	-	0	-	-	21	-	-	-	-	-	All
28	Iqbal	Kuwait	9	1999	8	2001	ICT	Unknown	HRP2	Yes	9	46	Febrile	Chloroquine or quinine	Non-ACT	?	240	-	-	82.1	-	-	-	62.4	-	34.9	-	-	-	-	-	-	-	-	None
29	Iqbal	Kuwait	9	1999	8	2001	ICT	Unknown	HRP2/pLDH (Pan)	Yes	9	46	Febrile	Chloroquine or quinine	Non-ACT	?	240	-	-	32.8	-	-	-	30.1	-	19.7	-	-	-	-	-	-	-	-	None
30	Iqbal	Kuwait	9	1999	8	2001	OptiMal	Unknown	pLDH (Pan)	No	9	46	Febrile	Chloroquine or quinine	Non-ACT	?	240	-	-	38.4	-	-	-	14.8	-	-	-	-	-	-	-	-	-	-	None
31	Rubio	Returned travellers	?	1998	?	1999	ParaSight-F	Unknown	HRP2	No	1	72	Returned travellers	Chloroquine	Non-ACT	?	169	-	-	-	-	-	-	-	50	-	-	-	-	-	-	-	-	-	None
32	Rubio	Returned travellers	?	1998	?	1999	OptiMal	Unknown	pLDH (Pan)	No	1	72	Returned travellers	Chloroquine	Non-ACT	?	169	-	-	-	-	-	-	-	-	-	50	-	-	-	-	-	-	-	None
33	Grandesso	Uganda	9	2011	1	2013	SD Bioline	05FK50-02-4	HRP2	No	0	5	Febrile	ACT (artesunate-amodiaquine)	ACT	1 to 1,999	73	-	92	88	-	75	-	71	-	58	-	37	23	16	7	-	-	-	None
34	Grandesso	Uganda	9	2011	1	2013	SD Bioline	05FK50-02-4	HRP2	No	0	5	Febrile	ACT (artesunate-amodiaquine)	ACT	2000 to 199,999	386	-	100	95	-	95	-	86	-	84	-	65	50	34	-	-	-	-	None
35	Grandesso	Uganda	9	2011	1	2013	SD Bioline	05FK50-02-4	HRP2	No	0	5	Febrile	ACT (artesunate-amodiaquine)	ACT	>200,000	59	-	100	100	-	100	-	96	-	91	-	80	80	43	-	-	-	-	None
36	Grandesso	Uganda	9	2011	1	2013	Carestart	G0111	HRP2	No	0	5	Febrile	ACT (artesunate-amodiaquine)	ACT	1 to 1,999	73	-	94	92	-	92	-	85	-	85	-	66	52	30	22	-	-	-	None
37	Grandesso	Uganda	9	2011	1	2013	Carestart	G0111	HRP2	No	0	5	Febrile	ACT (artesunate-amodiaquine)	ACT	2000 to 199,999	386	-	100	98	-	95	-	95	-	92	-	92	75	63	45	-	-	-	None
38	Grandesso	Uganda	9	2011	1	2013	Carestart	G0111	HRP2	No	0	5	Febrile	ACT (artesunate-amodiaquine)	ACT	>200,000	59	-	100	100	-	100	-	98	-	95	-	90	86	75	74	-	-	-	None
39	Grandesso	Uganda	9	2011	1	2013	Carestart	G0111	pLDH (Pan)	No	0	5	Febrile	ACT (artesunate-amodiaquine)	ACT	1 to 1,999	73	-	100	100	-	13	-	13	-	7	-	1	1	1	0	-	-	-	None
40	Grandesso	Uganda	9	2011	1	2013	Carestart	G0111	pLDH (Pan)	No	0	5	Febrile	ACT (artesunate-amodiaquine)	ACT	2000 to 199,999	386	-	100	100	-	26	-	26	-	0	-	0	0	0	0	-	-	-	None
41	Grandesso	Uganda	9	2011	1	2013	Carestart	G0111	pLDH (Pan)	No	0	5	Febrile	ACT (artesunate-amodiaquine)	ACT	>200,000	59	-	100	100	-	46	-	46	-	26	-	19	19	19	1	-	-	-	None
42	Singh	India	4	2004	8	2004	ParaHit	25977	HRP2	No	0**	99**	Febrile	Chloroquine or primaquine	Non-ACT	?	94	-	-	-	-	-	-	-	41.5	-	-	-	-	-	-	-	-	-	None
43	Mayxay	Thailand	11	1998	5	2000	Parasight-F	Unknown	HRP2	No	0**	99**	Severe and uncomplicated malaria cases	Artesunate (monotherapy or with azithromucine or lumefantrine)	Non-ACT	?	52	-	-	-	-	-	-	-	-	67	-	-	-	-	-	-	-	-	None
44	Humar	Returned travelers	1	1994	7	1995	Parasight-F	Unknown	HRP2	No	0**	99**	Returned travellers	?	Non-ACT	?	19	-	-	95	-	-	-	68	-	-	-	-	27	-	-	-	-	-	None
45	Schiff	Tanzania	?	?	?	?	Parasight-F	Unknown	HRP2	No	14	60	Febrile	Sulfadoxine-pyrimethamine	Non-ACT	?	40	-	97.5	50	30	25	20	20	10	10	-	-	-	-	-	-	-	-	None
46	Karbwang	Thailand/Myanmar	?	?	?	?	Parasight-F	Unknown	HRP2	No	0**	99**	Febrile [excluded if parasitemia > 5%]	Artemether	Non-ACT	?	72	55.6	69.4	-	-	-	-	52.1	-	25.4	-	-	-	-	-	-	-	-	Some
47	Fogg	Uganda	4	2005	6	2005	Paracheck	Unknown	HRP2	No	0**	99**	Febrile	ACT (artemether-lumefantrine)	ACT	<250,000	226	-	-	86.2	-	-	-	80.8	-	69.7	-	-	-	-	-	-	-	-	None
48	Fogg	Uganda	4	2005	6	2005	Vistapan	Unknown	pLDH (Pan)	No	0**	99**	Febrile	ACT (artemether-lumefantrine)	ACT	<250,000	221	-	-	36.1	-	-	-	23.4	-	8.9	-	-	-	-	-	-	-	-	None
49	Fogg	Uganda	4	2005	6	2005	Carestart	Unknown	pLDH (Pan)	No	0**	99**	Febrile	ACT (artemether-lumefantrine)	ACT	<250,000	230	-	-	42.5	-	-	-	27.6	-	9.5	-	-	-	-	-	-	-	-	None
50	Fogg	Uganda	4	2005	6	2005	Parabank	Unknown	pLDH (Pan)	No	0**	99**	Febrile	ACT (artemether-lumefantrine)	ACT	<250,000	204	-	-	17.8	-	-	-	8.9	-	4.6	-	-	-	-	-	-	-	-	None
51	Yadav	India	5	2011	4	2013	ParaHit	Unknown	HRP2	No	0.25	12	Febrile	Chroroquine & primaquine	Non-ACT	?	270	-	-	-	-	-	-	-	24.4	-	-	-	-	-	-	-	-	-	None
52	Nyunt	Myanmar	10	2010	12	2010	SD Bioline	Cat No 05FK60, Lot No 090007	HRP2	Yes	6	99**	Febrile	ACT (artemether-lumefantrine)	ACT	500 to 100,000	63	-	-	100	-	-	-	98.4	-	90.5	-	71.4	60.32	-	-	-	-	-	None
53	Nyunt	Myanmar	10	2010	12	2010	SD Bioline	Cat No 05FK60, Lot No 090007	pLDH (Pan)	Yes	6	99**	Febrile	ACT (artemether-lumefantrine)	ACT	500 to 100,000	63	-	-	28.6	-	-	-	12.7	-	4.8	-	4.8	3.17	-	-	-	-	-	None
54	Mharakurwa	Zimbabwe	5	<1997	6	<1997	ParaSight-F	Unknown	HRP2	No	0**	99**	Febrile	Chloroquine	Non-ACT	?	55	78	92	82	90	92	64	67	60	60	-	-	-	-	-	-	-	-	Some
55	Mueller	PNG	4	2000	1	2003	Optimal	Unknown	pLDH (Pan)	No	1.4	45	Febrile	Cotrimoxazole-rifampin-isoniazid or mefloquine or sulfadoxine-pyrimethamine	Non-ACT	?	255	-	31.4	-	-	-	-	20.8	-	2.5	-	-	-	-	-	-	-	-	None
56	Kilauzi	DRC	10	2012	12	2012	SD Bioline	Unknown	HRP2	Yes	0.25	4	Febrile	ACT (artesunate-amodiaquine)	ACT	?	103	-	-	100	-	-	-	100	-	95	-	86.2	84.1	-	-	-	-	-	None
57	Kilauzi	DRC	10	2012	12	2012	SD Bioline	Unknown	pLDH (Pan)	Yes	0.25	4	Febrile	ACT (artesunate-amodiaquine)	ACT	?	103	-	-	7.8	-	-	-	1.9	-	1	-	0	11.4	-	-	-	-	-	Some
58	Gerstl	Sierra Leone	3	2008	7	2008	Carestart	G0121	pLDH (Pan)	No	0	4	Febrile	ACT (artesunate-amodiaquine)	ACT	?	155	-	63.9	-	-	-	-	5.8	-	-	-	0.6	-	-	-	-	-	-	None
59	Nour	Sudan	9	2003	3	2004	Paracheck	Unknown	HRP2	No	0**	99**	Febrile	Chloroquine	Non-ACT	?	52	94.2	93.9	86	-	-	-	35	-	9.4	-	0	0	-	-	-	-	-	None
60	Nour	Sudan	9	2003	3	2004	Optimal	Unknown	pLDH (Pan)	No	0**	99**	Febrile	Chloroquine	Non-ACT	?	52	92.3	75.5	41.9	-	-	-	20	-	9.4	-	0	0	-	-	-	-	-	None
61	Kocharekar	India	5	2010	11	2011	Advantage MalCard	ACM15101	pLDH (Pan)	No	18	70	Febrile	?	ACT	?	101	-	-	-	-	-	-	14.9	-	7.92	-	-	-	-	-	-	-	-	None
62	Kocharekar	India	5	2010	11	2011	ParaHit	4000006623	HRP2	No	18	70	Febrile	?	ACT	?	101	-	-	-	-	-	-	37.3	-	58.41	-	-	-	-	-	-	-	-	All
63	Moody	Returned travellers	?	<2000	?	<2000	Optimal	Unknown	pLDH (Pan)	No	0**	99**	Returned travellers	?	Non-ACT	?	58	76	61	62	68	38	-	-	-	-	-	-	-	-	-	-	-	-	Some
64	Miller	Thailand	?	<2001	?	<2001	Optimal	Unknown	pLDH (Pan)	No	0**	99**	Febrile	?	Non-ACT	?	12	100	75	0	-	-	-	-	-	-	-	-	-	-	-	-	-	-	None
65	Wongsrichanalai	Thailand	1	1997	7	1997	ICT	Unknown	HRP2	No	0**	99**	Microscopy-confirmed *P. falciparum* patients	ACT (artesunate-mefloquine-primaquine)	ACT	?	35	-	70	-	-	-	-	47	-	44	-	-	-	-	-	-	-	-	None
66	Djimde	Mali	10	2009	12	2009	SD Bioline	05FK50	HRP2	No	0**	99**	Febrile	ACT (artemether-lumefantrine)	ACT	?	25	-	-	-	-	-	-	100	-	100	-	-	-	-	-	-	-	-	None
67	Djimde	Mali	10	2009	12	2009	SD Bioline	05FK60	HRP2/pLDH (Pan)	No	0**	99**	Febrile	ACT (artemether-lumefantrine)	ACT	?	25	-	-	-	-	-	-	100	-	100	-	-	-	-	-	-	-	-	None
																	**Total number of study groups reporting observations for each day:**	15	30	43	4	12	2	60	5	57	1	36	42	23	16	1	1	1	

"?" Represents unknown values; not reported in text

* Two different surveying periods were used, but only one aggregated pool of data was reported. The start and end dates of the two surveying periods are listed here with (1) and (2) as the first and second surveying period, respectively.

** Studies with ages listed as 0-99 did not report specific ages of subjects but confirmed that subjects of all ages were included.

† For the purposes of analysis, RDTs labelled "HRP2" are grouped as HRP2-only RDTs, and all other RDTs (including those in combination with HRP2) are grouped as other RDTs.

^ "Lines on same test" refers to study groups where combination RDTs were used but the result for the individual lines on each RDT were reported. Study groups from these publications feature twice with each result reported separately.

*** "Points omitted?" column refers to whether points were omitted due to increases in positive proportions. If labelled "None", all points were used in the analysis. If labelled "Some", one or more points were omitted from analysis due to small increases. If labelled "All", the entire row was omitted from analysis due to large increases.

ID	Reference
1	Kyabayinze DJ, Tibenderana JK, Odong GW, Rwakimari JB, Counihan H. Operational accuracy and comparative persistent antigenicity of HRP2 rapid diagnostic tests for Plasmodium falciparum malaria in a hyperendemic region of Uganda. Malar J 2008; 7: 221.
2	Mbabazi P, Hopkins H, Osilo E, Kalungu M, Byakika-Kibwika P, Kamya MR. Accuracy of two malaria rapid diagnostic tests (RDTS) for initial diagnosis and treatment monitoring in a high transmission setting in Uganda. Am J Trop Med Hyg 2015; 92: 530–6.
3	Mbabazi P, Hopkins H, Osilo E, Kalungu M, Byakika-Kibwika P, Kamya MR. Accuracy of two malaria rapid diagnostic tests (RDTS) for initial diagnosis and treatment monitoring in a high transmission setting in Uganda. Am J Trop Med Hyg 2015; 92: 530–6.
4	Kattenberg JH, Tahita CM, Versteeg IAJ, et al. Antigen persistence of rapid diagnostic tests in pregnant women in Nanoro, Burkina Faso, and the implications for the diagnosis of malaria in pregnancy. Trop Med Int Heal 2012; 17: 550–7.
5	Swarthout TD, Counihan H, Senga RKK, van den Broek I. Paracheck-Pf accuracy and recently treated Plasmodium falciparum infections: is there a risk of over-diagnosis? *Malar J* 2007; **6**: 58
6	Swarthout TD, Counihan H, Senga RKK, van den Broek I. Paracheck-Pf accuracy and recently treated Plasmodium falciparum infections: is there a risk of over-diagnosis? *Malar J* 2007; **6**: 58
7	Swarthout TD, Counihan H, Senga RKK, van den Broek I. Paracheck-Pf accuracy and recently treated Plasmodium falciparum infections: is there a risk of over-diagnosis? *Malar J* 2007; **6**: 58
8	Swarthout TD, Counihan H, Senga RKK, van den Broek I. Paracheck-Pf accuracy and recently treated Plasmodium falciparum infections: is there a risk of over-diagnosis? *Malar J* 2007; **6**: 58
9	Swarthout TD, Counihan H, Senga RKK, van den Broek I. Paracheck-Pf accuracy and recently treated Plasmodium falciparum infections: is there a risk of over-diagnosis? *Malar J* 2007; **6**: 58
10	Ly AB, Tall A, Perry R, et al. Use of HRP-2-based rapid diagnostic test for Plasmodium falciparum malaria: assessing accuracy and cost-effectiveness in the villages of Dielmo and Ndiop, Senegal. Malar J 2010; 9: 153
11	Houze S, Dicko Boly M, Le Bras J, Deloron P, Faucher J-F. PfHRP2 and PfLDH antigen detection for monitoring the efficacy of artemisinin-based combination therapy (ACT) in the treatment of uncomplicated falciparum malaria. Malar J 2009; 8: 211.
12	Houze S, Dicko Boly M, Le Bras J, Deloron P, Faucher J-F. PfHRP2 and PfLDH antigen detection for monitoring the efficacy of artemisinin-based combination therapy (ACT) in the treatment of uncomplicated falciparum malaria. Malar J 2009; 8: 211.
13	Houze S, Dicko Boly M, Le Bras J, Deloron P, Faucher J-F. PfHRP2 and PfLDH antigen detection for monitoring the efficacy of artemisinin-based combination therapy (ACT) in the treatment of uncomplicated falciparum malaria. Malar J 2009; 8: 211.
14	Houze S, Dicko Boly M, Le Bras J, Deloron P, Faucher J-F. PfHRP2 and PfLDH antigen detection for monitoring the efficacy of artemisinin-based combination therapy (ACT) in the treatment of uncomplicated falciparum malaria. Malar J 2009; 8: 211.
15	Houze S, Dicko Boly M, Le Bras J, Deloron P, Faucher J-F. PfHRP2 and PfLDH antigen detection for monitoring the efficacy of artemisinin-based combination therapy (ACT) in the treatment of uncomplicated falciparum malaria. Malar J 2009; 8: 211.
16	Houze S, Dicko Boly M, Le Bras J, Deloron P, Faucher J-F. PfHRP2 and PfLDH antigen detection for monitoring the efficacy of artemisinin-based combination therapy (ACT) in the treatment of uncomplicated falciparum malaria. Malar J 2009; 8: 211.
17	Mohammed H, Kassa M, Kebede A, Endeshaw T. Paracheck-pf® Test Versus Microscopy in the diagnosis of falciparum malaria in Arbaminch Zuria woreda of South Ethiopia. *Ethiop J Health Sci* 2012; **22**: 93–8.
18	Aydin-Schmidt B, Mubi M, Morris U, et al. Usefulness of Plasmodium falciparum-specific rapid diagnostic tests for assessment of parasite clearance and detection of recurrent infections after artemisinin-based combination therapy. Malar J 2013; 12: 349.
19	Aydin-Schmidt B, Mubi M, Morris U, et al. Usefulness of Plasmodium falciparum-specific rapid diagnostic tests for assessment of parasite clearance and detection of recurrent infections after artemisinin-based combination therapy. Malar J 2013; 12: 349.
20	Huong NM, Davis TME, Hewitt S, et al. Comparison of three antigen detection methods for diagnosis and therapeutic monitoring of malaria: a field study from southern Vietnam. Trop Med Int Health 2002; 7: 304–8.
21	Huong NM, Davis TME, Hewitt S, et al. Comparison of three antigen detection methods for diagnosis and therapeutic monitoring of malaria: a field study from southern Vietnam. Trop Med Int Health 2002; 7: 304–8.
22	Tjitra E, Suprianto SRI, Broom JMC, Currie BJ. Persistent ICT Malaria P . f / P . v Panmalarial and HRP2 Antigen Reactivity after Treatment of Plasmodium falciparum Malaria Is Associated with Gametocytemia and Results in False-Positive Diagnoses of Plasmodium vivax in Convalescence. J Clin Microbiol 2001; 39: 1025–31.
23	Tjitra E, Suprianto SRI, Broom JMC, Currie BJ. Persistent ICT Malaria P . f / P . v Panmalarial and HRP2 Antigen Reactivity after Treatment of Plasmodium falciparum Malaria Is Associated with Gametocytemia and Results in False-Positive Diagnoses of Plasmodium vivax in Convalescence. J Clin Microbiol 2001; 39: 1025–31.
24	Tjitra E, Suprianto SRI, Broom JMC, Currie BJ. Persistent ICT Malaria P . f / P . v Panmalarial and HRP2 Antigen Reactivity after Treatment of Plasmodium falciparum Malaria Is Associated with Gametocytemia and Results in False-Positive Diagnoses of Plasmodium vivax in Convalescence. J Clin Microbiol 2001; 39: 1025–31.
25	Tjitra E, Suprianto SRI, Broom JMC, Currie BJ. Persistent ICT Malaria P . f / P . v Panmalarial and HRP2 Antigen Reactivity after Treatment of Plasmodium falciparum Malaria Is Associated with Gametocytemia and Results in False-Positive Diagnoses of Plasmodium vivax in Convalescence. J Clin Microbiol 2001; 39: 1025–31.
26	Tjitra E, Suprianto SRI, Broom JMC, Currie BJ. Persistent ICT Malaria P . f / P . v Panmalarial and HRP2 Antigen Reactivity after Treatment of Plasmodium falciparum Malaria Is Associated with Gametocytemia and Results in False-Positive Diagnoses of Plasmodium vivax in Convalescence. J Clin Microbiol 2001; 39: 1025–31.
27	Tjitra E, Suprianto SRI, Broom JMC, Currie BJ. Persistent ICT Malaria P . f / P . v Panmalarial and HRP2 Antigen Reactivity after Treatment of Plasmodium falciparum Malaria Is Associated with Gametocytemia and Results in False-Positive Diagnoses of Plasmodium vivax in Convalescence. J Clin Microbiol 2001; 39: 1025–31.
28	Iqbal J, Siddique A, Jameel M, Hira PR. Persistent Histidine-Rich Protein 2 , Parasite Lactate Dehydrogenase , and Panmalarial Antigen Reactivity after Clearance of Plasmodium falciparum Monoinfection. J Clin Microbiol 2004; 42: 4237–41.
29	Iqbal J, Siddique A, Jameel M, Hira PR. Persistent Histidine-Rich Protein 2 , Parasite Lactate Dehydrogenase , and Panmalarial Antigen Reactivity after Clearance of Plasmodium falciparum Monoinfection. J Clin Microbiol 2004; 42: 4237–41.
30	Iqbal J, Siddique A, Jameel M, Hira PR. Persistent Histidine-Rich Protein 2 , Parasite Lactate Dehydrogenase , and Panmalarial Antigen Reactivity after Clearance of Plasmodium falciparum Monoinfection. J Clin Microbiol 2004; 42: 4237–41.
31	Rubio JM, Buhigas I, Subirats M, Baquero M, Puente S. Limited Level of Accuracy Provided by Available Rapid Diagnosis Tests for Malaria Enhances the Need for PCR-Based Reference Laboratories. 2001; 39: 2736–7.
32	Rubio JM, Buhigas I, Subirats M, Baquero M, Puente S. Limited Level of Accuracy Provided by Available Rapid Diagnosis Tests for Malaria Enhances the Need for PCR-Based Reference Laboratories. 2001; 39: 2736–7.
33	Grandesso F, Nabasumba C, Nyehangane D, et al. Performance and time to become negative after treatment of three malaria rapid diagnostic tests in low and high malaria transmission settings. Malar J 2016; 15:496.
34	Grandesso F, Nabasumba C, Nyehangane D, et al. Performance and time to become negative after treatment of three malaria rapid diagnostic tests in low and high malaria transmission settings. Malar J 2016; 15:496.
35	Grandesso F, Nabasumba C, Nyehangane D, et al. Performance and time to become negative after treatment of three malaria rapid diagnostic tests in low and high malaria transmission settings. Malar J 2016; 15:496.
36	Grandesso F, Nabasumba C, Nyehangane D, et al. Performance and time to become negative after treatment of three malaria rapid diagnostic tests in low and high malaria transmission settings. Malar J 2016; 15:496.
37	Grandesso F, Nabasumba C, Nyehangane D, et al. Performance and time to become negative after treatment of three malaria rapid diagnostic tests in low and high malaria transmission settings. Malar J 2016; 15:496.
38	Grandesso F, Nabasumba C, Nyehangane D, et al. Performance and time to become negative after treatment of three malaria rapid diagnostic tests in low and high malaria transmission settings. Malar J 2016; 15:496.
39	Grandesso F, Nabasumba C, Nyehangane D, et al. Performance and time to become negative after treatment of three malaria rapid diagnostic tests in low and high malaria transmission settings. Malar J 2016; 15:496.
40	Grandesso F, Nabasumba C, Nyehangane D, et al. Performance and time to become negative after treatment of three malaria rapid diagnostic tests in low and high malaria transmission settings. Malar J 2016; 15:496.
41	Grandesso F, Nabasumba C, Nyehangane D, et al. Performance and time to become negative after treatment of three malaria rapid diagnostic tests in low and high malaria transmission settings. Malar J 2016; 15:496.
42	Singh N, Mishra AK, Shukla MM, Chand SK, Bharti PK. Diagnostic and prognostic utility of an inexpensive rapid on site malaria diagnostic test (ParaHIT f) among ethnic tribal population in areas of high , low and no transmission in central India. 2005; 5: 1–5.
43	Mayxay M, Pukrittayakamee S, Chotivanich K, Looareesuwan S, White NJ. Persistence of Plasmodium falciparum malaria falciparum HRP-2 in successfully treated acute. Trans R Soc Trop Med Hyg 2001; 95: 179–82.
44	Humar A, Ohrt C, Harrington MA, Pillai D, Kain KC. ParasightF Test compared with the polymerase chain reaction and microscopy for the diagnosis of Plasmodium falciparum malaria in travelers. Am J Trop Med Hyg 1997; 56: 44–8.
45	Shiff CJ, Premji Z, Diseases I. The rapid manual ParaSighP-F falciparum infection test . A new diagnostic tool for Plasmodium falciparum infection. Trans R Soc Trop Med Hyg 1993; 87: 646–8.
46	Karbwang J, Tasanor O, Kanda T, et al. ParaSight-F Plasmodium test for the detection falciparum malaria. Trans R Soc Trop Med Hyg 1996; 90: 513–5.
47	Fogg C, Twesigye R, Batwala V, et al. Assessment of three new parasite lactate dehydrogenase (pan-pLDH) tests for diagnosis of uncomplicated malaria. Trans R Soc Trop Med Hyg 2008; 102: 25–31.
48	Fogg C, Twesigye R, Batwala V, et al. Assessment of three new parasite lactate dehydrogenase (pan-pLDH) tests for diagnosis of uncomplicated malaria. Trans R Soc Trop Med Hyg 2008; 102: 25–31.
49	Fogg C, Twesigye R, Batwala V, et al. Assessment of three new parasite lactate dehydrogenase (pan-pLDH) tests for diagnosis of uncomplicated malaria. Trans R Soc Trop Med Hyg 2008; 102: 25–31.
50	Fogg C, Twesigye R, Batwala V, et al. Assessment of three new parasite lactate dehydrogenase (pan-pLDH) tests for diagnosis of uncomplicated malaria. Trans R Soc Trop Med Hyg 2008; 102: 25–31.
51	Yadav RK, Singh MV, Singh DK, et al. Comparative study of RDTs vs microscopy for the diagnosis of malaria in children. J Evol Med Dent Sci 2014; 3: 6152–7.
52	Nyunt MH, Kyaw MP, Win KK, Myint KM, Nyunt KM. Field evaluation of HRP2 and pan pLDH-based immunochromatographic assay in therapeutic monitoring of uncomplicated falciparum malaria in Myanmar. 2013; 12:123.
53	Nyunt MH, Kyaw MP, Win KK, Myint KM, Nyunt KM. Field evaluation of HRP2 and pan pLDH-based immunochromatographic assay in therapeutic monitoring of uncomplicated falciparum malaria in Myanmar. 2013; 12:123.
54	Mharakurwa S, Shiff CJ. Post treatment sensitivity studies with the ParaSight-F test for malaria diagnosis in Zimbabwe. Acta Trop 1997; 66: 61–7.
55	Mueller I, Betuela I, Ginny M, Reeder JC, Genton B. The Sensitivity of the OptiMAL Rapid Diagnostic Test to the Presence of Plasmodium falciparum Gametocytes Compromises Its Ability To Monitor Treatment Outcomes in an Area of Papua New Guinea in which Malaria Is Endemic ⍰ 2007; 45: 627–30.
56	Kilauzi AL, Gaby J, Mulumba T, et al. SD Bioline malaria antigen Pf (HRP-2/ LHD) for assessing efficacy of artemisinin combination therapy against Plasmodium falciparum in pediatric patients in the Democratic Republic of the Congo. Pan Afr Med J 2015; 8688: 1–7.
57	Kilauzi AL, Gaby J, Mulumba T, et al. SD Bioline malaria antigen Pf (HRP-2/ LHD) for assessing efficacy of artemisinin combination therapy against Plasmodium falciparum in pediatric patients in the Democratic Republic of the Congo. Pan Afr Med J 2015; 8688: 1–7.
58	Gerstl S, Dunkley S, Mukhtar A, Smet M De, Baker S, Maikere J. Assessment of two malaria rapid diagnostic tests in children under five years of age , with follow- up of false-positive pLDH test results , in a hyperendemic falciparum malaria area, Sierra Leone. Mal. J. 2010; 9:28.
59	Nour B, Schallig H, Mens P, Elbushra S, Sa O, Mohamadani A. Use of Rapid Diagnostic Tests (RDTs) to monitor the efficacy of anti-malaria therapy in Gezira, Sudan- A prospective, assessor- blind comparative study. Int J Med 2009; 2: 257–62.
60	Nour B, Schallig H, Mens P, Elbushra S, Sa O, Mohamadani A. Use of Rapid Diagnostic Tests (RDTs) to monitor the efficacy of anti-malaria therapy in Gezira, Sudan- A prospective, assessor- blind comparative study. Int J Med 2009; 2: 257–62.
61	Kocharekar MM, Sarkar SS, Dasgupta D. Comparative Study of Modified Quantitative Buffy Coat and Two Rapid Tests in Comparison with Peripheral Blood Smear in Malaria Diagnosis in Mumbai , India. J Parasitol Res. 2014; 2014: 194651.
62	Kocharekar MM, Sarkar SS, Dasgupta D. Comparative Study of Modified Quantitative Buffy Coat and Two Rapid Tests in Comparison with Peripheral Blood Smear in Malaria Diagnosis in Mumbai , India. J Parasitol Res. 2014; 2014: 194651.
63	Moody A, Hunt-Cooke A, Gabbett E, Chiodini P. Performance of the OptiMAL malaria antigen capture dipstick for malaria diagnosis and treatment monitoring at the Hospital for Tropical Diseases, London. Br J Haematol 2000; 109: 891–4.
64	Miller RS, McDaniel P, Wongsrichanalai C. Following the course of malaria treatment by detecting parasite lactate dehydrogenase enzyme. Br J Haematol 2001; 113: 558–62.
65	Wongsrichanalai C, Chuanak N, Tulyayon S, Thanoosingha N. Comparison of a rapid field immunochromatographic test to expert microscopy for the detection of Plasmodium falciparum asexual parasitemia in Thailand. 1999; 73: 263–73.
66	Djimde AA, Maiga H, Sagara I, et al. Field assessment of SD bioline malaria species antigen detection by rapid diagnostic tests in Mali. J Parasitol Vector Biol 2016; 8.
67	Djimde AA, Maiga H, Sagara I, et al. Field assessment of SD bioline malaria species antigen detection by rapid diagnostic tests in Mali. J Parasitol Vector Biol 2016; 8.
