# Supplementary material for: How long do rapid diagnostic tests remain positive after anti-malarial treatment?
Source: Malar J. 2018 Jun 8;17:228. doi: 10.1186/s12936-018-2371-9 (PMC5994115; doi:10.1186/s12936-018-2371-9)
Supplement: Supplementary file 2 — Additional file 2. Bayesian survival model priors and likelihoods. [file 12936_2018_2371_MOESM2_ESM.pdf]

Additional File 2: Bayesian survival model priors and likelihoods

Priors

$\boldsymbol{\beta_i \sim N(0, \sigma_\beta)}$ for $\boldsymbol{i = 1, 2, \ldots, 12}$

$\boldsymbol{Z_j \sim N(0, \sigma_Z)}$ for $\boldsymbol{j = 1, 2, \ldots, 62}$

$\boldsymbol{\sigma_\beta \sim Exp(1)}$

$\boldsymbol{\sigma_Z \sim Exp(1)}$

$\boldsymbol{log\, c_k \sim N(log\, c_{k-1}, \frac{1}{10}(t_k - t_{k-1}))}$

Likelihoods

Given a survival curve $S(t)$, the likelihood of an individual being RDT positive at time t_{lower} and RDT negative at t_{upper} is

$$\mathrm{P}(t_{lower} < t < t_{upper}) = S(t_{lower}) - S(t_{upper})$$

For individuals who are still RDT positive at time of the end of study, t_{max} , is

$$P(t > t_{max}) = S(t_{max})$$
